# Software-Aided Versus Unassisted Fluoroscopy in Optimising Limb Length and Component Placement in Direct Anterior Total Hip Arthroplasty

**DOI:** 10.7759/cureus.92660

**Published:** 2025-09-18

**Authors:** Leina Suzuki, Selin Munir, Shuhei Hiyama, Hemant Pandit, Yash Wagh, Daevyd Rodda, Francis Connon

**Affiliations:** 1 Clinical Research, Medacta Australia Pty Ltd, Lane Cove, AUS; 2 School of Health, University of Sunshine Coast, Birtinya, AUS; 3 Research Department, Fortius Institute for Musculoskeletal Research, Birtinya, AUS; 4 Leeds Institute of Rheumatic and Musculoskeletal Medicine, University of Leeds, Leeds, GBR; 5 Department of Orthopaedics, Jichi Medical University, Tochigi, JPN; 6 Department of Orthopaedics, Sunshine Coast University Hospital, Birtinya, AUS

**Keywords:** direct anterior approach (daa), image intensifier, intra-operative fluoroscopy, myhip verifier, software-aided

## Abstract

Background

Intraoperative fluoroscopy has been the gold standard in direct anterior approach (DAA) total hip arthroplasty (THA) to assess the position of the components. Fluoroscopy could be aided by software to help surgeons plan and execute the THA optimally; however its clinical effectiveness has not been proven. This study assessed the use of fluoroscopy with intraoperative software to evaluate the ability of the software to accurately measure the component position and leg length discrepancy (LLD) in DAA.

Methods

Thirty-eight patients (39 procedures) were prospectively selected from consecutive patients treated using software-aided fluoroscopy (Group 1). Thirty-eight patients (38 procedures) were retrospectively enrolled into the control group (Group 2) from patients using intraoperative fluoroscopy without software analysis. Patients in both groups underwent THA using DAA. A mobile C-arm image intensifier unit was used to obtain images of the affected hip intraoperatively. In Group 1, the LLD and cup inclination were calculated intraoperatively by the software. The LLD, cup inclination and anteversion were measured for both groups using antero-posterior (AP) X-rays at six weeks postoperatively and were compared to the preoperatively planned targets.

Results

The mean postoperative LLD was significantly less in Group 1 when compared to Group 2 (2.2mm vs 4.6mm, p<0.01). In Group 1, 100% of hips (39/39) had a radiographic LLD within 10mm compared with 97% (37/38) in Group 2. The cup inclination and cup anteversion achieved within Group 1 were closer to the planned inclination and anteversion when compared to the control.

Conclusions

Intraoperative software analysis of fluoroscopy in THA aids accurate implantation of THA using the DAA approach with regard to leg length equality and acetabular component positioning.

## Introduction

Dislocation, along with infection, is the most common cause for revision in the first 11 years following a primary total hip arthroplasty (THA) [1]. Correctly positioned acetabular components play a crucial role in preventing dislocation, as well as other postoperative complications such as impingement, increased wear and reduced range of motion [2-6]. As such, positioning the implant in the correct alignment is essential for successful functional outcome and improved survivorship of implants in THA [7,8]. Although leg length discrepancy (LLD) may not be the most common reason for revision, a significant LLD can cause hip instability and dislocation. LLD also continues to be a major medicolegal concern in THA, being the single greatest cause of litigations [9-11]. As such, the ability to measure LLD and assess component position in real time during the surgery is necessary to optimise patient satisfaction and reduce the risks of complications after a THA.

It has become a common practice in direct anterior approach (DAA) THA to utilise C-arm fluoroscopy intraoperatively as it allows surgeons to assess the component positioning and, if necessary, alter planned acetabular component placement [12,13]. Additionally, fluoroscopy allows components to be positioned based on functional pelvic orientation as the patients can be positioned to recreate their patient-specific pelvic tilt [14-16]. By allowing for the pelvis to be levelled, the LLD can also be measured intraoperatively. Whilst fluoroscopy is considered practical and cost-effective, there is image distortion associated with its use due to the optical phenomenon seen with fluoroscopy, which can significantly impact how a surgeon interprets cup orientation and limb length [16,17]. Indeed, there is a learning curve, and with practice the surgeon can interpret the fluoroscopy images, although the time taken to master the interpretation can vary.

The software used in this study (MyHip Verifier, Medacta International SA, Castel San Pietro, Switzerland) requires a trained individual (not the surgeon) in the operating room to collate the images taken intraoperatively with the C-arm fluoroscopy. The software then calculates the acetabular component orientation and measures the leg length correction from its preoperative state in real time. The pre-implantation radiograph, taken whilst in supine in the operating theatre, is manually overlayed onto a radiograph taken with the trial components or immediately after the definitive components have been implanted. 

The aim of this study was to determine whether the accuracy of identifying the LLD and acetabular cup position is improved when intraoperative fluoroscopy is aided by software when compared to using unassisted fluoroscopy intraoperatively.

## Materials and methods

Study design

This study received institutional ethical review board approval from Prince Charles Hospital Human Research Ethics Committee (approval 80355). Between January 2021 and September 2021, consecutive patients who had THA operations by the two senior authors were included in the study. All participants were provided with details of the surgical treatment and provided consent for the procedure as part of standard practice of care. The requirement for individual consent to be part of the study was waived by the committee. However, participants were provided an opt-out consent form if they wished to be excluded from the study. Group 1 was defined as the software-aided group, where the THA was assisted with the MyHip Verifier software intraoperatively, and Group 2 was defined as the control, where a standard (unassisted) fluoroscopy was used. The patient demographics and surgery details have been summarised in Table 1. There were 39 hips (38 patients) that were prospectively enrolled into Group 1, where 49% were men. Group 2 consisted of 38 hips (38 patients) who were retrospectively enrolled; 42% were men. No patients from either cohort required revision or had experienced any complications.

**Table 1:** General and demographic data

		Software-aided (Group 1)	Control (Group 2)	P-value
Age of patients (years)		69.9±10.0	67.3±10.5	0.21
Body Mass Index (BMI)		29.6±4.8	28.2±3.6	0.15
Gender	Men	18	16	0.64
	Women	20	22	
Operative side	Left	20	13	0.13
	Right	17	25	
	Bilateral (patients)	1	0	

Surgical procedure

**Figure 1:**
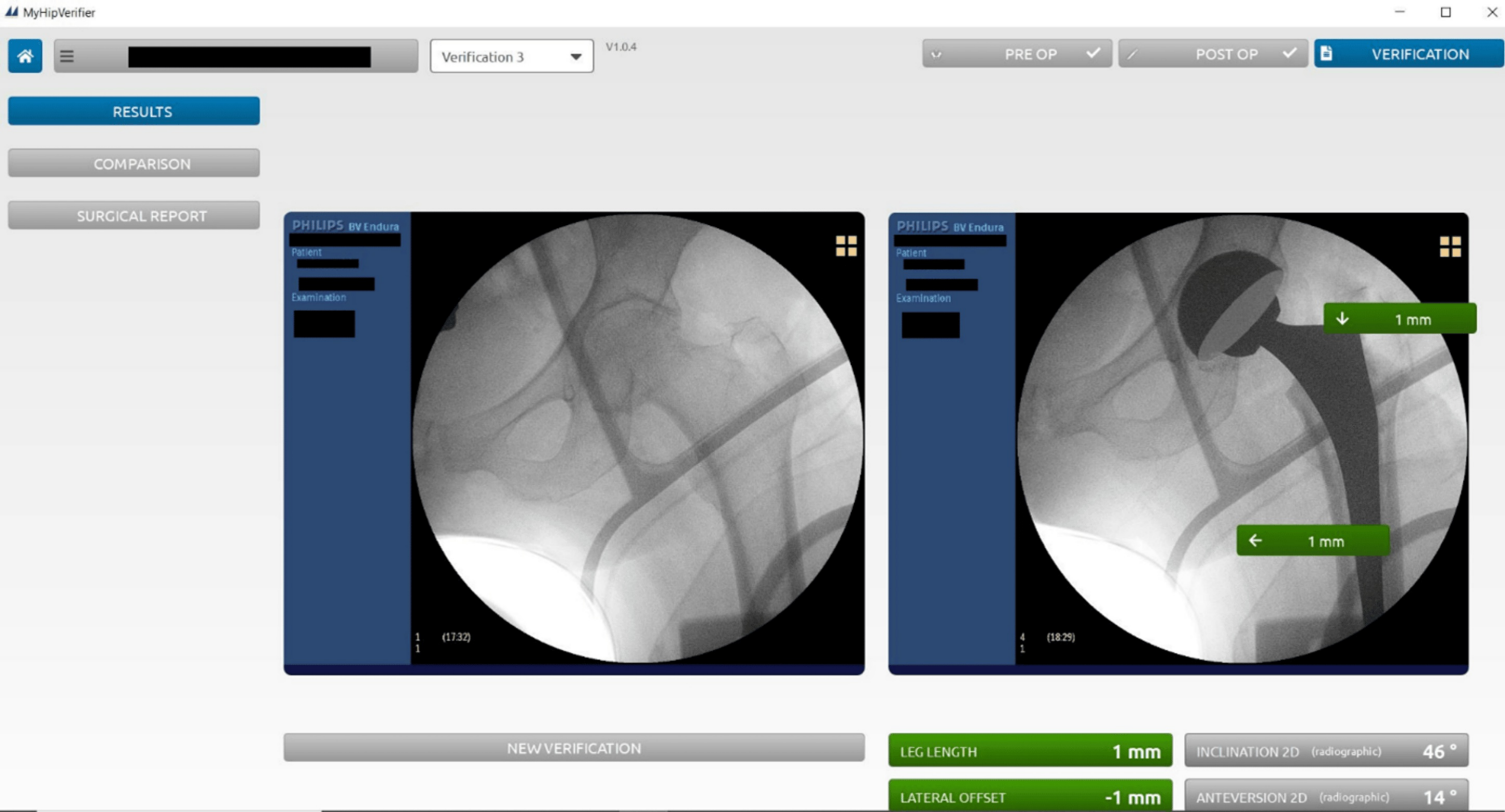
User interface of the MyHip Verifier Software displaying the baseline ‘preoperative’ image (left) and postoperative image with the trial components (right). The calculated measurements conducted by the software are also displayed.

All patients received a THA using a DAA. The implant position was templated preoperatively using a 3D plan to determine planned cup inclination and anteversion for both cohorts. The LLD was measured during the preoperative planning and the target leg length was planned with consideration of the contralateral side. The target cup inclination was 45°, based on an acceptable range of 35-55°, anteversion was planned to be within a range of 10°-30° for all patients. The patients were positioned supine on an operating table with an assisted device, allowing for a mobile C-arm image intensifier to be positioned to obtain X-ray images of the affected hip. In the operating room, a baseline intraoperative X-ray was first taken, with the leg positioned in 10° internal rotation. Once the baseline intraoperative image had been taken, the operating table and C-arm height were locked, with the position of the C-arm marked on the floor to ensure the remaining intraoperative X-rays would match the baseline image. The baseline image was then transferred from the C-arm to the software to be landmarked by a separate individual, while the investigator continued the surgery. Using the software, the pelvis axis of rotation and reference points were set manually. An intraoperative X-ray was taken after the trial cup had been implanted and then again after the trial stem had been implanted. Landmarks taken using the intraoperative image helped identify the acetabular cup edge. The implant position and LLD were verified prior to implantation of the definitive implants. Tests for impingement and instability were conducted in line with the tests used for a standard on-table direct anterior approach. The final implant position and LLD were measured and recorded using the software after the definitive implants were implanted, as shown in Figure 1.

Leg length, cup inclination and cup anteversion analysis

Standing antero-posterior (AP) X-rays taken at six weeks post-surgery for all cohorts were used to measure these parameters. The final limb length correction was achieved, and cup inclination was assessed by two independent evaluators and cup anteversion values by a single evaluator two times. Correction of preoperative LLD to within 10mm of the unoperated limb was considered an acceptable benchmark, as per literature [18]. To analyse the accuracy of the cup position using the software, the absolute difference in cup inclination was compared between the target alignment, inclination calculated by the software, and postoperative measurements. The anteversion between the two groups was also compared.

The change in leg length from its preoperative state was measured as the perpendicular distance from the inter-teardrop line to the most medial tip of the lesser trochanter using a standing AP X-ray taken prior to the surgery date and six weeks post-operation. In Group 1, the intraoperative images with the trial components were manually overlayed onto the baseline intraoperative x-ray for the software to calculate the change in leg length from its preoperative state. In Group 2, LLD was evaluated intraoperatively with the use of a transverse metal rod to compare the distances between a transverse line drawn across the ischial tuberosities and the lesser trochanters.

The cup inclination was calculated as the angle between the greater diameter of the cup face and a parallel line to the ischial tuberosities using the AP X-rays taken at six weeks post-operation. Similar to the leg length measurement, the cup inclination in Group 1 was calculated by the software once the trial component image was overlayed onto the preoperative image by the separate individual. Three points were taken to reference the cup edge that was used to calculate the diameter of the cup face. The change in values was compared to the preoperative plan.

The cup anteversion was calculated as the angle with the methods of Widmer [19]. The short axis of the ellipse (S) and the total length (TL) of the projected cross-section of the component along the short axis were measured. This method shows a linear correlation for values of S/TL between 0.2 and 0.6. Thus, determining acetabular cup anteversion can be reduced to computing the S/TL ratio and looking for the corresponding anteversion angle in a table or graph. The change in values was compared to the preoperative plan.

Statistical analysis

The mean measurements were calculated at all timepoints and analysed for statistical significance using Microsoft Excel (Microsoft, Redmond, WA, USA), where statistical significance was set at <0.05. The Pearson’s Chi-square test was used to evaluate differences between categorical values, and the Mann-Whitney U-test was used to evaluate differences between continuous and ordinal data of the two groups. Pearson’s correlation test was used to assess the correlation between preoperative and postoperative LLD.

## Results

Leg length discrepancy

The mean LLD measured at six weeks postoperatively was significantly less in Group 1 when compared to Group 2 (2.2±3.0mm vs 4.6±3.2mm; p<0.01). One hundred percent (39/39) of hips from Group 1 and 97% (37/38) of hips from Group 2 had an LLD of within 10mm (p=0.31).

**Figure 2:**
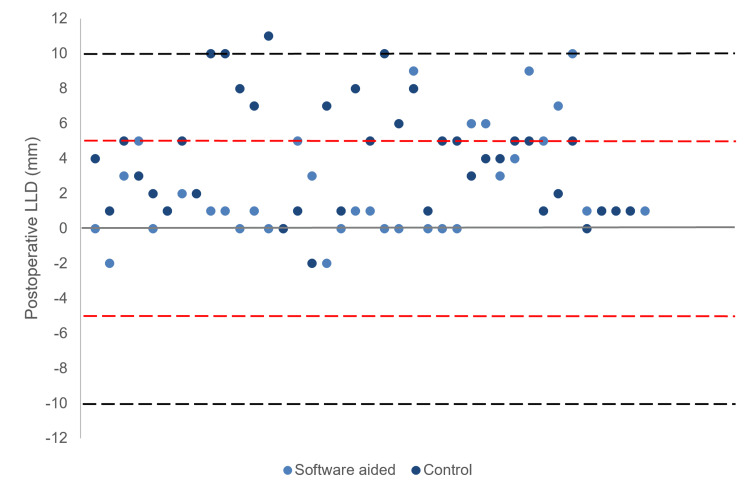
Postoperative Leg Length Discrepancy (LLD). Black dotted line indicates range within ±10mm. Red dotted line indicates range within ±5mm.

Group 1 had a lower proportion of patients outside the postoperative LLD of 5mm in comparison to Group 2 (15% vs 32%; p=0.09). The spread of LLD between the groups can be seen in Figure 2. 

Cup inclination

**Figure 3:**
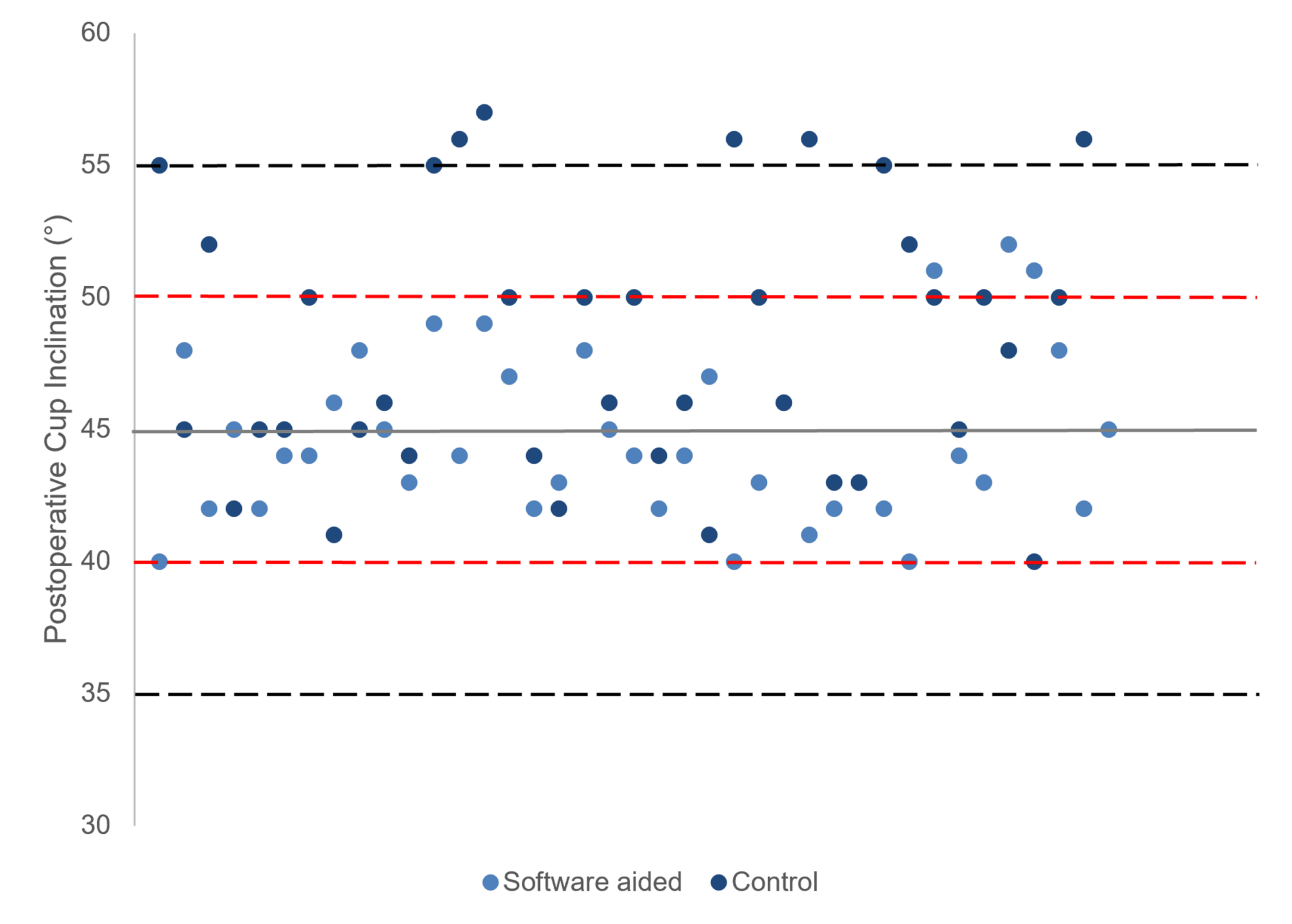
Cup inclination measured at six weeks postoperatively. Black dotted line indicates range within ±10° from planned inclination. Red dotted line indicates range within ±5° from planned inclination.

The mean absolute difference in cup inclination achieved compared to the planned inclination was significantly less in Group 1, in comparison to the control cohort (2.6±1.8° vs 4.6±3.8°, p=0.05). The mean absolute difference in cup inclination as measured using the software and six weeks postoperatively was 0.4±0.9°. Figure 3 displays the distribution of cup inclinations of the two groups. Zero percent (0/39) of hips from Group 1 had a cup inclination outside the target by ±10°, in comparison to 13% (5/38) from Group 2 (p=0.02). There were 8% (3/39) of hips from Group 1 and 26% (10/38) from Group 2 that had a cup inclination outside the target by ±5° (p=0.03).

Cup anteversion

The mean absolute difference in cup anteversion achieved compared to the planned anteversion was significantly less in Group 1, in comparison to the control cohort (1.9±1.5° vs 3.4±2.7°, p<0.01). There were 5.1% (2/39) of hips from Group 1 that had a cup anteversion outside the target by ±5°, in comparison to 23.7% (9/38) from Group 2 (p=0.02).

## Discussion

Fluoroscopy has been the gold standard for DAA-THA for its ability to verify the component positioning intraoperatively. Jennings et al. [20] conducted a study to assess whether fluoroscopy improved the accurate placement of the acetabular component when compared to non-fluoroscopic guided DAA-THA. The study showed 88% of cups positioned with fluoroscopy to be within ±10° from the planned inclination, in comparison to the non-fluoroscopy cohort of 72%. Similarly, Beamer et al. [13] demonstrated that THA guided with fluoroscopy results in an accurate component position when compared to freehand technique, where the use of fluoroscopy resulted in 74% of cups being positioned within their chosen threshold of 30° to 45° inclination. In this current study, Group 2 showed consistent outcomes, where 87% of components positioned with fluoroscopy alone had an inclination within ±10° from the planned position (45°). However, whilst the results from Group 2 support the accuracy of fluoroscopy, this study found that the accuracy of the cup alignment can be further improved with software-aided fluoroscopy, with 100% of cups from Group 1 positioned within ±10° threshold, and only 8% outside a ±5° range.

The reliability and practicality of fluoroscopy alone in accurately determining component position have been questioned, with issues relating to the impact of the optimal phenomenon being raised. The distortion caused by electromagnetic fields has been shown to significantly impact how limb length is seen intraoperatively whilst parallax can cause misinterpretation of cup anteversion as a result of objects appearing to be displaced [16,17]. The positioning of the pelvis to reproduce the preoperative standing radiograph, as well as the radiographic projection of the C-arm also plays a crucial role in the accurate and correct positioning of the components [14,21].

The inaccuracy in component measurements between fluoroscopy alone and AP X-rays has been demonstrated by Delagrammaticas et al. [22], where measurements taken using different projections and fluoroscopic techniques were compared to measurements taken from postoperative AP X-rays. The study found that both fluoroscopic hip and pelvis had underestimated the cup inclination relative to postoperative AP X-rays, with a mean difference of 2.75° and 0.79°, respectively. In this current study, we did not find a significant difference between the cup inclination measured using the postoperative AP X-rays and the measurements from the MyHip Verifier software, with a mean difference of 0.4°. None of the patients in either group experienced any dislocation. Whether they perceived LLD, this was not assessed in this study. The results from Group 1 demonstrated a smaller difference in comparison to both fluoroscopic hip and pelvis from Delagrammatica et al.’s cohort. As the independent reviewers from the current study and Delagrammatica et al.’s study had passed the learning curve of fluoroscopy, the superior results from Group 1 can suggest that the accuracy of fluoroscopy can be improved when the measurements are calculated by an integrated software. The improved accuracy of software-aided fluoroscopy could also be due to the consistency in the images taken in Group 1, where extra care was taken to ensure that the projection was identical in both the baseline and post-implantation intraoperative images.

The correct measurement of postoperative inclinations and anteversion is crucial for this assessment. Several radiological methods have been described for recording the anteversion of the acetabular component on plain radiographs. But few studies have compared the reliability and accuracy of these methods [23,24]. Nomura et al. reported the reliability of five methods of assessing anteversion (Lewinnek et al. [4], Widmer [19], Liaw et al. [25], Pradhan et al. [26], and Woo and Morrey [27]) and compared the accuracy of these methods with those of CT measurements using the same definition and reference plane [28]. The author reported that Widmer’s method was not significantly different from CT measurements. Therefore, we chose Widmer’s method as the best for assessing anteversion on plain radiographs. In our study, the intraclass correlation coefficient for measurements on plain radiographs was 0.894 (95% Confidence Interval: 0.839 to 0.931) for the intra-observer reliability of anteversion with Widmer’s method.

While component positioning plays a crucial role in preventing complications after surgery, perception of a leg length discrepancy remains the main cause of litigation and thus patient dissatisfaction in THA [10]. LLD is universally perceived when there is a discrepancy of greater than 10mm [18]. In DAA-THA, the conventional method of evaluating LLD intraoperatively is by measuring the distance between reference points on the pelvis and femur or by overlaying a printed fluoroscopic image with trial implants on a preoperative image [10,29,30]. However, it is known that these methods of measuring LLD intraoperatively are limited by beam asymmetry and changes in the position of the femur [31]. This was evident in the current study, where patients from Group 2 had a greater LLD when compared to those in Group 1. While the senior author of this study was well experienced in both techniques, the ability to measure and therefore correct for LLD intraoperatively could have assisted the software-aided group to have fewer outliers than the control group. O’Leary et al. [31] also found that software-aided fluoroscopy improved the accuracy in executing the planned limb length, where the mean difference between planned and achieved leg length was significantly less in the software-assisted fluoroscopy cohort when compared to fluoroscopy alone. 

Robotic-assisted THA has increased in popularity as a solution to validate implant position and measure LLD intraoperatively, with the ability to provide accurate, reproducible THA with fewer outliers [32-37]. Kanawade et al. [37] found that with robotic-assisted THA, 12% of hips had inclination outliers between 5° to 10° and 0% with outliers greater than 10°. Whilst it has been demonstrated that robotic-assisted THA can assist in an accurate execution of the planned alignment, the results presented in literature are comparable to the software-aided cohort of this study. Likewise, in a study by El Bitar et al. [38], where the accuracy in measuring LLD using robotic-assisted, fluoroscopy-guided anterior and conventional posterior approaches were compared, the study found that while no patients had a LLD of 10mm or more, there was a higher percentage of outliers over 5mm in the robotic-assisted cohort than the other methods. In addition, the overall cost and significant modification to the surgical workflow associated with robotic-assisted THA have limited its widespread use [39-44]. Robotic-assisted THA is also associated with a steep learning curve ranging from 12-35 cases from the added technical complexity [44-46].

Software-aided fluoroscopy provides the advantage of robotic-assisted THA, with the ability to assist surgeons in executing planned alignment in theatres with similar accuracy. However, in comparison to robotic-assisted THA, the use of the software-aided fluoroscopy requires minimal alteration to the surgical workflow from a standard fluoroscopic THA. There are no checkpoint registrations necessary, thereby eliminating the need for additional invasive pins whilst minimising the time commitment required from the operating surgeon. Aside from the consistency required for the setup of the C-arm, there are minimal changes to the operating surgeon’s workflow as the software is handled by an external experienced individual. Consequently, the software-aided fluoroscopy does not increase operative time. While robotic-assisted devices are required to be within the sterile field of the operating room and take up a significant amount of intraoperative space, the software-aided fluoroscopy is much smaller and does not need to be within the sterile field of the room, as it can be processed at any location.

Limitations of this study include the relatively small cohort and the lack of long-term clinical outcomes assessed. The software-aided cohort was performed by a single surgeon (FC), whilst the unassisted fluoroscopy cohort was performed by two surgeons. This may have introduced potential bias to the surgical techniques. While a single trained individual navigated the software in majority of the cases, this was not controlled in this study. All individuals who had navigated the software had received training on how to use the software. However, user variability may have affected the results from this study. Pelvic tilt can change between functional positions, which can affect the functional orientation of the cup position. As the acetabular component position was compared from radiographs taken in supine intraoperatively to standing radiographs taken postoperatively, this may have resulted in some differences in the measured acetabular cup orientation.

## Conclusions

Whilst image intensification alone cannot guard us against leg length inequality and cup placement inaccuracy, this study demonstrated the use of software-aided fluoroscopy in allowing for an accurate positioning of the components to the planned cup inclination and anteversion as well as minimising LLD.
